# Interferon α-2b spray shortened viral shedding time of SARS-CoV-2 Omicron variant: An open prospective cohort study

**DOI:** 10.3389/fimmu.2022.967716

**Published:** 2022-08-05

**Authors:** Nan Xu, Jinjin Pan, Li Sun, Cuimei Zhou, Siran Huang, Mingwei Chen, Junfei Zhang, Tiantian Zhu, Jiabin Li, Hong Zhang, Yufeng Gao

**Affiliations:** ^1^ Department of Infectious Diseases, the First Affiliated Hospital of Anhui Medical University, Hefei, China; ^2^ Department of Endocrinology, the First Affiliated Hospital of Anhui Medical University, Hefei, China; ^3^ Departerment of Emergency, Anhui Public Health Clinical Centre, Hefei, China

**Keywords:** SARS-CoV-2 Omicron variant, COVID-19, IFN α-2b spray, viral shedding time, cohort study

## Abstract

**Background:**

The Omicron SARS-CoV-2 variant has spread quickly worldwide due to its effects on virus transmission and vaccine effectiveness. Interferon(IFN) has been shown to have a protective effect against SARS-CoV because of its broad antiviral activity. This study aimed to analyze the treatment effects of IFN α-2b spray in virus clearance of the Omicron SARS-CoV-2 variant.

**Methods:**

We examined the effectiveness and safety of IFN α-2b spray in Shanghai, China, with participants infected with the Omicron SARS-CoV-2 variant in an open, prospective cohort study from April 16th to May 5th, 2022.

**Results:**

A total of 871 confirmed patients were enrolled in this study. Four hundred and thirteen patients were allocated to the IFN α-2b spray group, and 458 patients were allocated to the control group. The viral shedding time was significantly different between experimental group and control group (11.90 vs.12.58, *P <*0.05). In the experimental group, the median administration time since the first positive test for SARS-CoV-2 was three days, ranging from 0 to 15 days. There was no obvious adverse effect associated with the spray of IFN α-2b. The univariate Cox regression analysis revealed that the administration time since the first positive test ≤3 days was a protective factor associated with viral shedding time (HR 0.81 95% CI 0.74-0.87, *P <*0.05). Subgroup analysis showed that the viral shedding time was 10.41 (4.00-16.00) days in the ≤3 days group, which was significantly less than that in the control group (12.58, 95% CI: 7.00-19.15, *P <*0.0001) and in the >3 days group (13.56, 95%CI: 7.00-22.25, *P <*0.0001).

**Conclusions:**

IFN α-2b spray shortened the viral shedding time of the Omicron SARS-CoV-2 variant when administrated within three days since the first positive test for SARS-CoV-2.

## Introduction

Currently, the Omicron outpaces the other variants of SARS-CoV-2 to be the dominant circulating strain, sweeping across the world (1). Over 500,000 local Omicron infections have been reported in China between 1 March and 22 April 2022, with the majority occurring in Shanghai (about 93%) (2). The major Omicron sub-lineages that prevail among the local novel coronavirus pneumonia(COVID-19) outbreaks in China are BA.1 and BA.2 (3–5). Paxlovid (nirmatrelvir/ritonavir) was authorized by the Chinese National medical products administration in February 12, 2022 for cases with mild to moderate COVID-19. However, the potential for significant drug-drug interactions, the high cost and the low-accessibility limit clinical use. Globally, there are an increasing number of cases and deaths and very limited treatment options, so new effective antiviral drugs are urgently needed.

Considering that type I interferons (IFNs) inhibit the replication of both DNA and RNA viruses at different stages of their replication cycles and effect on activating immune cell populations to clear infections, type I IFNs are directly antiviral agents (6). Based on its character of broad antiviral activity, IFN has been shown to exert a protective effect against SARS-CoV infection (7). However, patients with Covid-19 who received IFN treatment had little effect, as indicated by their overall mortality, the start of ventilation, and the length of their hospital stay in multiple clinical studies (8–10). The possible reason was that SARS-CoV-2 was capable of avoiding or disabling many of interferon’s effects (11). Nevertheless, a recent investigation revealed that Omicron variant has a lower ability to withstand host cell interferon responses. Further study showed that sequence variations in the SARS-CoV-2 IFN antagonists nsp3, nsp12, nsp13, nsp14, M protein, the nucleocapsid protein, and/or ORF3a may contribute to these differences (12, 13). As Omicron variant possesses a substantially enhanced IFN sensitivity, IFNs represent a promising option for the treatment of Omicron patients. Although a lot of meaningful exploration has been made, evidence of IFN effectiveness is mixed. Furthermore, the optimal route of administration and timing of IFN therapy to treat SARS-CoV-2 is not well documented.

Moreover, Nasal epithelium is thought to be one of the main entry points for SARS-CoV-2. The high transmissibility of SARS-CoV-2 is attributed to nasal epithelial tropism and efficient virus release from the nasopharynx. However, the main entrance of SARS-CoV-2, Angiotensin-converting enzyme 2 (ACE2), was expressed at very low protein levels in respiratory and olfactory epithelial cells. Another host factor, neuropilin-1 (NRP1) has been demonstrated as an entrance for SARS-CoV-2 infection (14). NRP1 represented as an ACE2 potentiating factor by promoting the interaction of the virus with ACE2 (15). A recent study analyzed the receptor-ligand interaction and found that the NRP1 coreceptor pathway may increase the infectivity of the Omicron variant of SARS-CoV-2 (16). Nasal cells mount a robust innate antiviral response to SARS-CoV-2 dominated by paracrine IFN-I/III signaling. Upon exposure to exogenous IFN-I/III, these cells undergo a profound antiviral response (17). A new study by Imperial College London showed that Omicron replicates rapidly in human primary airway cultures, enabling Omicron to infect more cells in the respiratory epithelium, allowing it to be more infectious at lower exposure doses and resulting in enhanced intrinsic transmissibility (18). Similarly, another study identified that Omicron variant replicates more rapidly in the respiratory tract than all other SARS-CoV-2 variants, but less efficiently in the lungs, which may explain the reduced severity of Omicron that is now being reported in epidemiological studies (19). As the highest viral loads are detectable in the upper respiratory tract, reducing infectious viruses in the nasopharynx could lower viral shedding and, consequently, transmission by infected individuals (20).

IFN spray could act on respiratory epithelium, and directly exerts antiviral activity. According to the study from Gao, using IFN α-2b for spray could effectively prevent respiratory infections caused by influenza viruses, para-influenza viruses, and adenoviruses (21). Therefore, IFN spray could be a potential prophylactic and therapeutic agent against SARS-CoV-2 Omicron variant. This study aimed to analyze the treatment effects of IFN α-2b spray in virus clearance of Omicron SARS-CoV-2 variant in an open, prospective cohort.

## Methods

### Patients and study design

**Figure 1:**
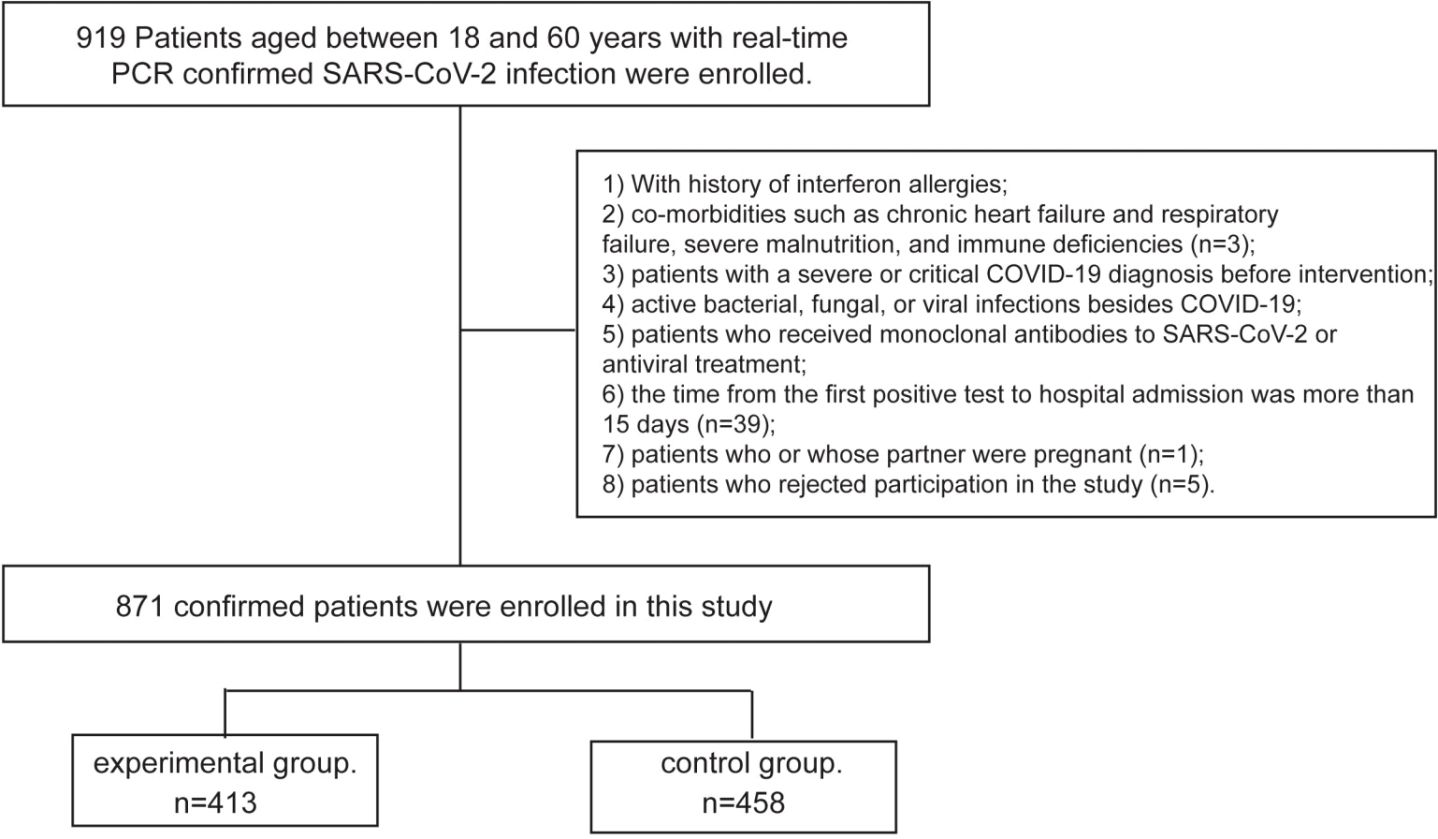
The flowchart of the study. Screening, enrolment and random classification of patients.

A total of 871 confirmed patients from Shanghai Temporary Hospital (Chongming District, Shanghai) were enrolled in this study from April 16th to May 5th, 2022. Four hundred and thirteen patients were allocated to the IFN α-2b spray group, and 458 patients were allocated to the control group (**Figure 1**). None of the asymptomatic or mild participants underwent blood tests, limited by the temporary hospital. The study was registered in the ClinicalTrials.gov (Registry NO. ChiCTR2200058790) and has been approved by the ethic committee of the first affiliated hospital of Anhui medical university (PJ-2022-0408). All participants provided informed consent before enrollment and drug administration.

This open, prospective cohort study aims to evaluate the safety and viral shedding time (real-time PCR Ct value >35 for both ORF1ab and N gene) of IFN α-2b spray in treating Omicron SARS-CoV-2 variant from April 16th to May 5th, 2022. Patients aged between 18 and 60 years, with real-time PCR confirmed SARS-CoV-2 infection were enrolled. Exclusion criteria: 1) With history of IFN allergies; 2) co-morbidities such as chronic heart failure and respiratory failure, severe malnutrition, and immune deficiencies; 3) patients with a severe or critical COVID-19 diagnosis before intervention; 4) active bacterial, fungal, or viral infections besides COVID-19; 5) patients who received monoclonal antibodies to SARS-CoV-2 or antiviral treatment; 6) the time from the first positive test to hospital admission was more than 15 days; 7) patients who or whose partner were pregnant, nursing, or likely to become pregnant; 8) patients who rejected participation in the study.

### Interventions

Complete medical history was taken, including demographic information, chronic disease history, symptoms of COVID-19 illness and vaccination status at the baseline after receiving consent from every participant. In addition to an essential clinical assessment and examination, appropriate protective measures were taken for all participants. The participants were randomized into two groups with the control group and the experimental group.

In the experimental group, recombinant human IFN α-2b were sprayed on the patients’ posterior pharyngeal wall, bilateral tonsils and oral lesions every 6 hours for seven days (3 sprays/time, about 1.2 million IU/day, ANKE Biotechnology (Group) Co., Ltd., HEFEI, CHINA). After spraying, diet and water were prohibited for 15 minutes. The control group did not receive IFN α-2b spray. All of the participants received symptomatic treatment based on their clinical manifestations, including non-steroidal anti-inflammatory drugs, cough mixtures and traditional Chinese medicine.

### Study definitions

Time to viral clearance was the primary outcome. SARS-CoV-2 RNA was tested daily in respiratory specimens from all patients until discharge. The nucleic acid test negative conversion was defined as two consecutive negative tests (Ct value >35 for the ORF1ab and N gene). The viral shedding time was defined as the duration from the first positive nucleic acid test to the date of the first negative test (in two consecutive, more than 24 hours apart). For patients still shedding virus at the end of the study, the time from the date of confirmed diagnosis to the final follow-up date of May 15th, 2022 was used for the calculation of viral shedding time.

### Statistical analysis

Continuous variables were expressed as medians (Range) or means (Standard Deviation, SD) and compared using a non-parametric test. Categorical variables were expressed as numbers (%) and compared by the χ² test or Fisher’s exact tests. The viral shedding time was compared between the two groups in both the primary and subgroup analyses. Hazard ratio (HR) and 95% confidence interval were calculated by Cox regression. A two-sided *P <*0.05 was considered statistically significant. Statistical analysis was performed using IBM SPSS 23.0 (IBM, Armonk, NY, USA).

## Results

Participant characteristics were generally similar between the two groups (**Table 1**) except for the sex ratio. The proportion of males in the experimental group (76.3%) was higher than that in the control group (67.2%). The average age was similar for both groups. There were 10 (2.4%) and 13 (2.8%) patients in the two groups who were obese. The experimental and control groups did not differ in smoking. Fewer cases of chronic diseases were observed in the experimental group (13.8% vs. 17.2%) with no significance. Other chronic diseases included stable chronic bronchitis, asthma, hypothyroidism, and chronic hepatitis B also had no significant difference. The most frequently reported symptoms were fever (45.0% vs. 36.0%) and cough (44.6% vs. 45.4%) in both groups. There were no significant differences between vaccination status in the two groups, and both groups had high vaccination rates (97.6% vs. 97.4%, *P* =0.852). There were 261 and 244 participants who received the booster dose, respectively. In the experimental group, the median administration time since the first positive test for SARS-CoV-2 was three days, ranging from 0 to 15 days. There was no obvious adverse effect associated with spray of IFN α-2b.

**Table 1:** The clinical characteristics of enrolled patients.

	Experimental(N=413)	Control(N=458)	P-value
Age, Mean± SD, years	39.0 ± 10.3	39.5 ± 10.3	0.463
Gender, n (%)			0.003
Female	98 (23.7)	150 (32.8)	
Male	315 (76.3)	308 (67.2)	
Obesity, n (%)			0.701
Normal	403 (97.6)	445 (97.2)	
Obesity	10 (2.4)	13 (2.8)	
Smoking, n (%)			0.165
Non-smoking	373 (90.3)	400 (87.3)	
Heavy smoking	40 (9.7)	58 (12.7)	
Chronic diseases, n (%)			0.162
Non-chronic diseases	356 (86.2%)	379 (82.8%)	
Chronic diseases	57 (13.8%)	79 (17.2%)	
Hypertension	48 (11.6)	68 (14.8)	
Diabetes	10 (2.4)	15 (3.3)	
Other	5 (1.2)	7 (1.5)	
Clinical classification, n(%)			0.690
Asymptomatic	185 (44.8)	199 (43.4)	
Symptomatic	228 (55.2)	259 (56.6)	
fever	186 (45.0)	165 (36.0)	
cough	184 (44.6)	208 (45.4)	
Sputum production	141 (34.1)	153 (33.4)	
Sore throat	128 (31.0)	156 (34.1)	
loss of gustation	25 (6.1)	37 (8.1)	
loss of olfaction	12 (2.9)	14 (3.1)	
Vaccination, n (%)			0.852
Unvaccinated	10 (2.4)	12 (2.6)	
Vaccinated	403 (97.6)	446 (97.4)	
Partially vaccinated	18 (4.5)	31 (7.0)	
Full vaccination	124 (30.8)	171 (38.3)	
Booster	261 (64.8)	244 (54.7)	
Admission time since the first positive test 0.120			
Median (range), days	3 (0-15)	3 (0-15)	
Mean ± SD, days	4.63 ± 3.94	3.86 ± 3.17	
Administration time since the first positive test			
≤3 days, n(%)	217 (52.5)	/	
>3 days, n(%)	196 (47.5)	/	
Median (range), days	3 (0-15)	/	
Mean ± SD, days	4.6 ± 3.9	/	

**Figure 2:**
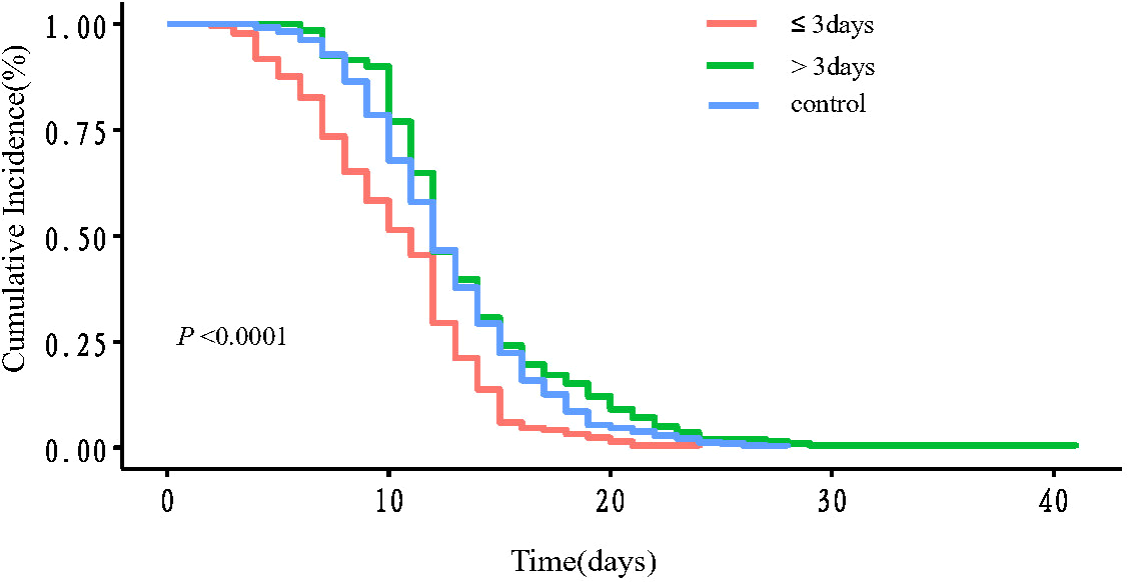
The Kaplan-Meier curve of the viral shedding time. Red line: ≤3 days group; green line: >3days groups; blue line: control group. *P <*0.0001 between ≤3 days group and control group; *P* =0.0176 between >3 days group and control group; *P <*0.0001 between ≤3 days group and >3 days group.

The univariate Cox regression analysis revealed that the administration time since the first positive test ≤3 days was a protective factor associated with the viral shedding time (**Table 2**, HR 0.81 95% CI 0.74-0.87, *P* <0.05). The viral shedding time was significantly different between experimental group and control group (11.90(5.00-20.00) vs.12.58(7.00-19.15), *P* = 0.024, **Table 3**). According to the median administration time since the first positive test for SARS-CoV-2, the experimental group was divided into ≤3 days group and>3 days group. Subgroup analysis showed that the viral shedding time was 10.41 (4.00-16.00) days in the ≤3 days group and 13.56 (7.00-22.25) days in the >3 days group (*P* <0.0001, **Table 3**). The subgroup analyses for vaccination, gender, obesity (BMI ≥30), heavy smoking (20 or more cigarettes per day), symptoms were performed. The effect of IFN α-2b spray on virus clearance was significant among vaccinated, non-obese, smoking, and symptomatic patients (**Table 3**). The above results and the Kaplan-Meier curves indicated that viral shedding resolved sooner in individuals prescribed IFN α-2b spray within three days of onset. The differences were statistically significant *(P* <0.0001, **Figure 2**).

**Table 2:** The hazard ratios, two-sided 95% confidence intervals, and P value were estimated with the use of Cox regression with the baseline stratification factors as covariates.

	Adjusted Hazard Ratio	*P* value
Administration time since the first positive test		
≤3 days	0.81 (0.74, 0.87)	0.000
>3 days	1.20 (1.02, 1.43)	0.031
Gender		
Male	0.96 (0.82,1.12)	0.540
Age	0.99 (0.99,1.00)	0.019
Obesity	1.44 (0.95,2.18)	0.088
Heavy smoking	0.97 (0.79,1.20)	0.805
Asymptomatic	1.00 (0.87,1.14)	0.980
Vaccination	0.83 (0.55,1.27)	0.397
Partially vaccinated	0.84 (0.55,1.29)	0.428
Full vaccination	1.03 (0.77,1.38)	0.836
Booster	1.03 (0.89,1.18)	0.741
Chronic diseases	0.87 (0.72,1.05)	0.137

**Table 3:** Main groups and subgroups analysis of the differences of the viral shedding time.

Main groups and subgroups(95% CI)	IFN(n=413)	Control(n=458)	*P*-value
Main groups 11.90 (5.00-20.00) 12.58 (7.00-19.15)			0.024
Administration time since the first positive test			
≤3 days	10.41 (4.00-16.00) n=217	12.58 (7.00-19.15) n=458	<0.0001
>3 days	13.56 (7.00-22.25) n=196	12.58 (7.00-19.15) n=458	0.077
Vaccination			
Unvaccinated	11.80 (5.90-16.55) n=10	14.33 (9.55-22.15) n=12	0.192
Vaccinated	11.90 (5.00-20.00) n=403	12.53 (7.00-19.00) n=446	0.038
Partially vaccinated	10.33 (4.70-15.00) n=18	13.16 (8.00-19.00) n=31	0.013
Full vaccination	11.92 (5.15-20.00) n=124	12.49 (7.00-19.00) n=171	0.254
Booster	12.00 (5.00-21.00) n=261	12.48 (7.00-21.85) n=244	0.238
Gender			
Female	11.78 (5.00-20.15) n=98	12.47 (8.00-18.55) n=150	0.210
Male	11.93 (8.99-20.00) n=315	12.63 (6.00-21.00) n=308	0.056
Obesity			
Non-obesity	11.95 (3.00-20.00) n=403	12.60 (7.00-19.80) n=445	0.033
Obesity	9.80 (4.25-14.55) n=10	11.69 (7.00-14.60) n=13	0.205
Smoking			
Non-smoking	12.00 (5.00-20.40) n=373	12.49 (7.00-19.00) n=400	0.126
Heavy smoking	10.98 (4.00-15.20) n=40	13.22 (6.00-24.00) n=58	0.011
Symptoms			
Asymptomatic	11.95 (4.20-20.00) n=185	12.38 (6.00-19.00) n=199	0.375
Symptomatic	11.86 (5.35-20.00) n=228	12.73 (7.00-21.00) n=259	0.021

## Discussion

From February 26th to May 5th, 2022, there were 55,131 cumulative confirmed cases and 562,863 cumulative asymptomatic cases reported in the Omicron variant of SARS-CoV-2 epidemic wave (22). The emerging studies show that the Omicron variant became milder than the previous variants, the trend of increasing cases and admissions waves shifted with a higher and quicker peak but fewer patients were admitted to hospital, less clinically severe illnesses (23, 24). However, a recent study published in JAMA revealed that all-cause excess mortality in Massachusetts during the first eight weeks of the Omicron period was more than that during the entire 23-week Delta period (25). It presumably reflects a higher mortality product (i.e., a moderately lower infection fatality rate multiplied by a far higher infection rate). A predictive model study from China showed that immunity induced by the March 2022 vaccination campaign would not be sufficient to prevent an Omicron wave. The study also showed that the Omicron wave would cause a projected intensive care unit peak demand of 15.6 times the existing capacity and cause approximately 1.55 million deaths (26). As previously reported, the Omicron caused more infections but less severe ones or deaths, while constant outbreaks and a large population base still put a tremendous amount of strain on the system.

Some new drugs such as Paxlovid (nirmatrelvir/ritonavir) are developing and being tested in clinical trials, but still hard to widely used due to the high cost and side effects. Therefore, it is urgent to develop a simple and effective anti-viral drug for combating the Omicron variant of SARS-CoV-2 pandemics. In this study, all participants were asymptomatic and mild cases. IFN α-2b spray significantly accelerated the viral shedding by 2-3 days when applied within three days since the first positive test for SARS-CoV-2. In addition, more than 97% of cases in experimental and control groups received the vaccine, which suggested that IFN α-2b spray might benefit people who have already been vaccinated. Subgroup analysis revealed that vaccinated participants cleared viral infection faster regardless of when the first injection occurred. Notably, the same effect was observed in non-obese, smoking and symptomatic cases.

Innate immunity, in particular IFN-I, is the first line of defense against viral infection. IFN-I has an essential role in the pathogenesis of COVID-19 (27–29). Even though rapid induction of type I IFNs prevents viral propagation, a sustained increase in the levels of type I IFNs in the late phase of the infection results in aberrant inflammation and poor clinical outcome (29–32). A study from Domizio et al. showed that the cyclic GMP-AMP synthase (cGAS)–stimulator of interferon genes (STING) pathway, which controls immunity to cytosolic DNA, was a critical driver of aberrant type I IFN responses in COVID-19 (33). It has been reported that early administration of therapeutic IFN could correct the imbalanced IFN response with excessive cytokine production caused by repressed type I IFN expression in critically ill COVID-19 patients (34). However, ACE2 has been demonstrated as a type I and III interferon-stimulated gene in human airway epithelial cells (35), which suggested that IFN may promote viral entry and replication in those cells. A multicenter cohort study has shown no association of early IFN use with CT scan improvement in survived patients, and late IFN use was associated with slower CT scan improvement (36). Similarly, we found that early use of IFN α-2b spray shortened viral shedding time, whereas delayed use may lead to prolonged viral shedding time. (**Table 3**, administration time since the first positive test 13.56 (7.00-22.25) vs. 12.58(7.00-19.15)).

Unlike other big proteins or molecules, Type I IFNs have been widely used as an anti-viral agent for a long time. Type I IFNs act through ubiquitously expressed IFN-α/β receptors (type I IFN receptor 1, IFNAR1 and IFNAR2), which are associated with tyrosine kinase 2 (TYK2) and Janus kinase 1 (JAK1), respectively (37). As the IFNAR receptors are generally widely expressed, the type I IFNs have a broad range of target cells, except red blood cells, phagocytes and kidney cells (38). However, the immunomodulatory action of IFN-α causes the release of a series of cytokines, including TNF-α, IL-1, IL-2, IL -6, and IFN-γ, resulting in a cytokine storm that leads to adverse reactions such as fever, muscle soreness, chills and other transient flu-like symptoms (39, 40). In our study, we choose the aerosol instead of intramuscular injection to avoid the side effect. The application of IFN α-2b spray had several advantages. First of all, the drug is commercially available, making it easier to apply than subcutaneous injections or atomized inhalations. Second, because the spray treatment can target the respiratory system directly, there is no need for systemic distribution. Third, as mentioned above, the use of IFN α-2b spray in this study did not lead to noticeable side effects. Finally, in contrast with atomization inhalation, IFN α-2b spray avoids droplet and aerosol transmission risks. However, there was a limitation in this study, the difference of viral shedding time between experimental and control group of unvaccinated patients had no significance. This may due to limited number of unvaccinated participants enrolled in this study.

In conclusion, our study is the first to evaluate the clinical function of IFN α-2b spray, which was an inexpensive, easily available, few side effects drug to the Omicron SARS-CoV-2 variant. Furthermore, IFN α-2b spray shortened the viral shedding time, and the administration time was within three days since the first positive test for SARS-CoV-2. However, the results of our study need to be further validated in other research before being clinically used in the future.

## Data availability statement

The raw data supporting the conclusions of this article will be made available by the authors, without undue reservation.

## Ethics statement

The studies involving human participants were reviewed and approved by The ethic committee of the first affiliated hospital of Anhui Medical University(PJ-2022-0408). The patients/participants provided their written informed consent to participate in this study.

## Author contributions

The study was designed and supervised by YG. The manuscript was written by NX, JP, SH. The data analysis was performed by LS, JZ, TZ. All authors were involved in critical revision of manuscript. All authors contributed to the article and approved the submitted version.

## Funding

This research was supported by the project of emergency scientific research of COVID-19 of Anhui Province (2022e07020078).

## Acknowledgments

We are grateful to all the patients who volunteered for this trial, as well as the personnel at the study sites. The authors thank Dr. Jiang Li for giving helpful comments on preparing manuscript. Study drug interferon α-2b spray was donated by Anhui ANKE Biotechnology (Group) Co., Ltd.

## Conflict of interests

The authors declare that the research was conducted in the absence of any commercial or financial relationships that could be construed as a potential conflict of interest.

## Publisher’s note

All claims expressed in this article are solely those of the authors and do not necessarily represent those of their affiliated organizations, or those of the publisher, the editors and the reviewers. Any product that may be evaluated in this article, or claim that may be made by its manufacturer, is not guaranteed or endorsed by the publisher.

## References

[1] Del Rio C Omer SB Malani PN. Winter of omicron-the evolving covid-19 pandemic. Jama (2022) 327(4):319–20. doi: 10.1001/jama.2021.24315 34935863

[2] The State Council Information Office PRCA. Press Conference Held on Situation Regarding Strict Prevention and Control of Covid-19 Epidemic. Available from:http://www.gov.cn/xinwen/gwylflkjz193/index.htm.

[3] Zhang D Wu S Ren Z Sun Y Dou X Feng Z. A local cluster of omicron variant covid-19 likely caused by internationally mailed document - Beijing municipality, China, January 2022. China CDC Wkly (2022) 4(14):302–4. doi: 10.46234/ccdcw2022.031 PMC9008263 35433094

[4] Li K Zheng Z Zhao X Zeng Q Zhou T Guo Q. An imported case and an infected close contact of the omicron variant of sars-Cov-2 - guangdong province, China, December 13, 2021. China CDC Wkly (2022) 4(5):96–7. doi: 10.46234/ccdcw2021.265 PMC8837459 35186377

[5] Guo Q Ruhan A Liang L Zhao X Deng A Hu Y. An imported case of Ba.2 lineage of omicron variant covid-19 - guangdong province, China, December 28, 2021. China CDC Wkly (2022) 4(5):98–9. doi: 10.46234/ccdcw2022.001 PMC8837461 35186378

[6] Wang BX Fish EN. Global virus outbreaks: Interferons as 1st responders. Semin Immunol (2019) 43:101300. doi: 10.1016/j.smim.2019.101300 31771760 PMC7128104

[7] Haagmans BL Kuiken T Martina BE Fouchier RA Rimmelzwaan GF van Amerongen G. Pegylated interferon-alpha protects type 1 pneumocytes against sars coronavirus infection in macaques. Nat Med (2004) 10(3):290–3. doi: 10.1038/nm1001 PMC7095986 14981511

[8] Pan H Peto R Henao-Restrepo AM Preziosi MP Sathiyamoorthy V Abdool Karim Q. Repurposed antiviral drugs for covid-19 - interim who solidarity trial results. New Engl J Med (2021) 384(6):497–511. doi: 10.1056/NEJMoa2023184 33264556 PMC7727327

[9] Jagannathan P Andrews JR Bonilla H Hedlin H Jacobson KB Balasubramanian V. Peginterferon lambda-1a for treatment of outpatients with uncomplicated covid-19: A randomized placebo-controlled trial. Nat Commun (2021) 12(1):1967. doi: 10.1038/s41467-021-22177-1 33785743 PMC8009873

[10] Kalil AC Mehta AK Patterson TF Erdmann N Gomez CA Jain MK. Efficacy of interferon beta-1a plus remdesivir compared with remdesivir alone in hospitalised adults with covid-19: A double-bind, randomised, placebo-controlled, phase 3 trial. Lancet Respir Med (2021) 9(12):1365–76. doi: 10.1016/s2213-2600(21)00384-2 PMC8523116 34672949

[11] Oh SJ Shin OS. Sars-Cov-2-Mediated evasion strategies for antiviral interferon pathways. J Microbiol (2022) 60(3):290–9. doi: 10.1007/s12275-022-1525-1 PMC8817151 35122601

[12] Bojkova D Widera M Ciesek S Wass MN Michaelis M Cinatl J Jr. Reduced interferon antagonism but similar drug sensitivity in omicron variant compared to delta variant of sars-Cov-2 isolates. Cell Res (2022) 32(3):319–21. doi: 10.1038/s41422-022-00619-9 PMC8781709 35064226

[13] Bojkova D Rothenburger T Ciesek S Wass MN Michaelis M Cinatl J Jr. Sars-Cov-2 omicron variant virus isolates are highly sensitive to interferon treatment. Cell Discovery (2022) 8(1):42. doi: 10.1038/s41421-022-00408-z 35538050 PMC9087166

[14] Daly JL Simonetti B Klein K Chen KE Williamson MK Antón-Plágaro C. Neuropilin-1 is a host factor for sars-Cov-2 infection. Science (2020) 370(6518):861–5. doi: 10.1126/science.abd3072 PMC7612957 33082294

[15] Wang HB Zhang H Zhang JP Li Y Zhao B Feng GK. Neuropilin 1 is an entry factor that promotes ebv infection of nasopharyngeal epithelial cells. Nat Commun (2015) 6:6240. doi: 10.1038/ncomms7240 25670642 PMC4339892

[16] Baindara P Roy D Mandal SM Schrum AG. Conservation and enhanced binding of sars-Cov-2 omicron spike protein to coreceptor neuropilin-1 predicted by docking analysis. Infect Dis Rep (2022) 14(2):243–9. doi: 10.3390/idr14020029 PMC9024780 35447881

[17] Hatton CF Botting RA Dueñas ME Haq IJ Verdon B Thompson BJ. Delayed induction of type I and iii interferons mediates nasal epithelial cell permissiveness to sars-Cov-2. Nat Commun (2021) 12(1):7092. doi: 10.1038/s41467-021-27318-0 34876592 PMC8651658

[18] Peacock TP Brown JC Zhou J Thakur N Newman J Kugathasan R. The sars-Cov-2 variant, omicron, shows rapid replication in human primary nasal epithelial cultures and efficiently uses the endosomal route of entry. bioRxiv (2022). doi: 10.1101/2021.12.31.474653

[19] Hui KPY Ho JCW Cheung MC Ng KC Ching RHH Lai KL. Sars-Cov-2 omicron variant replication in human bronchus and lung ex vivo. Nature (2022) 603(7902):715–20. doi: 10.1038/s41586-022-04479-6 35104836

[20] Bentley K Stanton RJ. Hydroxypropyl methylcellulose-based sprays effectively inhibit in vitro sars-Cov-2 infection and spread. Viruses (2021) 13(12):2345. doi: 10.3390/v13122345 34960612 PMC8705245

[21] Gao L Yu S Chen Q Duan Z Zhou J Mao C. A randomized controlled trial of low-dose recombinant human interferons alpha-2b spray to prevent acute viral respiratory infections in military recruits. Vaccine (2010) 28(28):4445–51. doi: 10.1016/j.vaccine.2010.03.062 PMC7115383 20394720

[22] commission Smh. Daily Briefing on Covid-19 in Shanghai. Available from: http://wsjkw.sh.gov.cn/xwfb/20220506/c682814657024377a49c7bc5745847d4.html.

[23] Jassat W Abdool Karim SS Mudara C Welch R Ozougwu L Groome MJ. Clinical severity of covid-19 in patients admitted to hospital during the omicron wave in south Africa: A retrospective observational study. Lancet Glob Health (2022) 10(7):e961-9. doi: 10.1016/s2214-109x(22)00114-0 35597249 PMC9116895

[24] Modes ME Directo MP Melgar M Johnson LR Yang H Chaudhary P. Clinical characteristics and outcomes among adults hospitalized with laboratory-confirmed sars-Cov-2 infection during periods of B.1.617.2 (Delta) and B.1.1.529 (Omicron) variant predominance - one hospital, California, July 15-September 23, 2021, and December 21, 2021-January 27, 2022. MMWR Morb Mortal Wkly Rep (2022) 71(6):217–23. doi: 10.15585/mmwr.mm7106e2 PMC8830624 35143466

[25] Faust JS Du C Liang C Mayes KD Renton B Panthagani K. Excess mortality in Massachusetts during the delta and omicron waves of covid-19. Jama (2022) 328(1):74–6. doi: 10.1001/jama.2022.8045 PMC9257575 35594035

[26] Cai J Deng X Yang J Sun K Liu H Chen Z. Modeling transmission of sars-Cov-2 omicron in China. Nat Med (2022) 28(7):1468–75. doi: 10.1038/s41591-022-01855-7 PMC9307473 35537471

[27] Lucas C Wong P Klein J Castro TBR Silva J Sundaram M. Longitudinal analyses reveal immunological misfiring in severe covid-19. Nature (2020) 584(7821):463–9. doi: 10.1038/s41586-020-2588-y PMC7477538 32717743

[28] Nienhold R Ciani Y Koelzer VH Tzankov A Haslbauer JD Menter T. Two distinct immunopathological profiles in autopsy lungs of covid-19. Nat Commun (2020) 11(1):5086. doi: 10.1038/s41467-020-18854-2 33033248 PMC7546638

[29] Park A Iwasaki A. Type I and type iii interferons - induction, signaling, evasion, and application to combat covid-19. Cell Host Microbe (2020) 27(6):870–8. doi: 10.1016/j.chom.2020.05.008 PMC7255347 32464097

[30] Hadjadj J Yatim N Barnabei L Corneau A Boussier J Smith N. Impaired type I interferon activity and inflammatory responses in severe covid-19 patients. Science (2020) 369(6504):718–24. doi: 10.1126/science.abc6027 PMC7402632 32661059

[31] Lee JS Park S Jeong HW Ahn JY Choi SJ Lee H. Immunophenotyping of covid-19 and influenza highlights the role of type I interferons in development of severe covid-19. Sci Immunol (2020) 5(49):eabd1554. doi: 10.1126/sciimmunol.abd1554 32651212 PMC7402635

[32] Galani IE Rovina N Lampropoulou V Triantafyllia V Manioudaki M Pavlos E. Untuned antiviral immunity in covid-19 revealed by temporal type I/Iii interferon patterns and flu comparison. Nat Immunol (2021) 22(1):32–40. doi: 10.1038/s41590-020-00840-x 33277638

[33] Domizio JD Gulen MF Saidoune F Thacker VV Yatim A Sharma K. The cgas-sting pathway drives type I ifn immunopathology in covid-19. Nature (2022) 603(7899):145–51. doi: 10.1038/s41586-022-04421-w PMC8891013 35045565

[34] Blanco-Melo D Nilsson-Payant BE Liu WC Uhl S Hoagland D Møller R. Imbalanced host response to sars-Cov-2 drives development of covid-19. Cell (2020) 181(5):1036–45.e9. doi: 10.1016/j.cell.2020.04.026 32416070 PMC7227586

[35] Ziegler CGK Allon SJ Nyquist SK Mbano IM Miao VN Tzouanas CN. Sars-Cov-2 receptor Ace2 is an interferon-stimulated gene in human airway epithelial cells and is detected in specific cell subsets across tissues. Cell (2020) 181(5):1016–35.e19. doi: 10.1016/j.cell.2020.04.035 32413319 PMC7252096

[36] Wang N Zhan Y Zhu L Hou Z Liu F Song P. Retrospective multicenter cohort study shows early interferon therapy is associated with favorable clinical responses in covid-19 patients. Cell Host Microbe (2020) 28(3):455–64.e2. doi: 10.1016/j.chom.2020.07.005 32707096 PMC7368656

[37] Uzé G Schreiber G Piehler J Pellegrini S. The receptor of the type I interferon family. Curr Top Microbiol Immunol (2007) 316:71–95. doi: 10.1007/978-3-540-71329-6_5 17969444

[38] de Weerd NA Nguyen T. The interferons and their receptors–distribution and regulation. Immunol Cell Biol (2012) 90(5):483–91. doi: 10.1038/icb.2012.9 PMC7165917 22410872

[39] Taylor JL Grossberg SE. The effects of interferon-alpha on the production and action of other cytokines. Semin Oncol (1998) 25(1 Suppl 1):23–9. 9482537

[40] Kirkwood JM Bender C Agarwala S Tarhini A Shipe-Spotloe J Smelko B. Mechanisms and management of toxicities associated with high-dose interferon Alfa-2b therapy. J Clin Oncol (2002) 20(17):3703–18. doi: 10.1200/jco.2002.03.052 12202672

